# Comparative neuropsychiatric safety signals of tacrolimus versus cyclosporine in solid organ transplantation: ten-year FAERS pharmacovigilance study

**DOI:** 10.3389/fimmu.2026.1795626

**Published:** 2026-03-27

**Authors:** Bassem A. Almalki, Khalid A. Alamer

**Affiliations:** College of Pharmacy, Imam Abdulrahman Bin Faisal University, Dammam, Saudi Arabia

**Keywords:** calcineurin inhibitors, tacrolimus, cyclosporine, FAERS, neuropsychiatric toxicity, solid organ transplantation

## Abstract

**Introduction:**

Neuropsychiatric toxicity is a recognized complication of calcineurin inhibitor (CNI) therapy; however, large-scale comparative assessments across tacrolimus immediate-release (IR), once-daily LCP-tacrolimus (LCPT), and cyclosporine remain limited.

**Methods:**

We performed a decade-long pharmacovigilance study using the FDA Adverse Event Reporting System (FAERS) database from 2015 to 2025 to characterize the spectrum, seriousness, temporal trends, and drug-specific reporting signals of neuropsychiatric adverse events (AEs) in transplant recipients in whom tacrolimus IR, LCPT, or cyclosporine was listed as a suspect agent. Events were categorized as neurological, psychiatric, or combined neuropsychiatric. Disproportionality was assessed using reporting odds ratios (RORs) and proportional reporting ratios (PRRs).

**Results:**

A total of 5,437 neuropsychiatric AE reports were identified, involving tacrolimus IR (71.9%), cyclosporine (23.7%), and LCPT (4.5%). Most reports were neurological (74.1%), followed by psychiatric (17.1%) and combined neuropsychiatric events (8.8%). Serious outcomes were frequently reported (92.5%), including hospitalization (52.1%) and reported death (16.2%). Combined neuropsychiatric events had the highest hospitalization rate (62.1%); however, such outcomes should be interpreted in the context of complex clinical illness and treatment exposure rather than as directly attributable to CNI-related neuropsychiatric toxicity alone. Tremor demonstrated elevated reporting disproportionality for tacrolimus IR (ROR 1.97; PRR 1.78) and LCPT (ROR 2.42; PRR 1.97). In contrast, cyclosporine showed no tremor signal but demonstrated elevated disproportionality for encephalopathy (ROR 1.73; PRR 1.45), insomnia (ROR 1.87; PRR 1.76), and anxiety (ROR 1.50; PRR 1.48). Reporting increased from 2015 to 2019 and remained elevated through 2025.

**Conclusion:**

These findings suggest distinct drug-specific neuropsychiatric reporting patterns among CNIs in FAERS. However, given the inherent limitations of spontaneous reporting systems and differences in real-world utilization across CNIs, these findings should be interpreted as hypothesis-generating pharmacovigilance signals rather than evidence of causation, incidence, or comparative risk. They highlight the potential clinical relevance of neuropsychiatric tolerability when individualizing CNI selection, monitoring, and formulation strategies, and support further integration of pharmacovigilance data with pharmacokinetic and registry-level evidence to improve individualized risk assessment.

## Introduction

Calcineurin inhibitors (CNIs), principally tacrolimus and cyclosporine, are the cornerstone of maintenance immunosuppression in solid organ transplantation and have markedly reduced acute rejection, translating into substantial improvements in short-term graft survival and patient outcomes (1). However, these benefits are offset by a narrow therapeutic index and a broad adverse-effect profile encompassing nephrotoxicity, cardiovascular and metabolic complications, and neurologic and neuropsychiatric toxicity (2).

CNI-related neurotoxicity is common and clinically heterogeneous. Across transplant populations, approximately 25–31% of tacrolimus-treated recipients develop some form of neurotoxic manifestation, with about 20% experiencing “mild” symptoms such as tremor, headache, insomnia, vertigo, dysesthesias, and mood change (3). For cyclosporine, neurotoxicity has been reported in up to 50% of patients, particularly in the early post-exposure period and at higher doses (4). In pooled analyses of studies with >100 patients, the incidence of tremor was ≈21.5% with tacrolimus and ≈17% with cyclosporine, with even higher tremor rates (≈30–42%) in kidney and bone marrow transplant cohorts (5). At the severe end of the spectrum, CNIs can precipitate seizures, encephalopathy, posterior reversible encephalopathy syndrome (PRES), psychosis, delirium, and other acute confusional states, which may lead to intensive care admission, prolonged hospitalization, or permanent neurological sequelae and typically occur within a broader, clinically complex setting rather than as isolated causes of death (6). When clinically significant neurotoxicity occurs, it often necessitates CNI dose reduction, temporary interruption, or conversion to an alternative regimen, with potential consequences for rejection risk and long-term allograft outcomes.

Comparative data suggest that tacrolimus may carry a higher burden of neurologic and neuropsychiatric complications than cyclosporine. Reviews of randomized and observational transplant studies consistently describe higher rates of tremor, paresthesia, and insomnia with tacrolimus than with cyclosporine (5). Case series and small cohorts further indicate that seizures, PRES, and psychotic syndromes occur more frequently in tacrolimus-treated patients, especially early after transplantation and in the setting of supratherapeutic concentrations or interacting medications (7). Mechanistically, the more pronounced neurotoxicity of tacrolimus has been attributed to greater lipophilicity and central nervous system penetration, higher pharmacokinetic variability with steep peak concentrations, and a higher susceptibility to clinically relevant drug–drug interactions via CYP3A and P-glycoprotein pathways (8). Nonetheless, the available evidence is fragmented, often underpowered, and heterogeneous in terms of study design, outcome definitions, and follow-up duration, limiting precise estimates of relative risk.

In addition to molecule-level differences between tacrolimus and cyclosporine, tacrolimus is available as immediate-release capsules and once-daily extended-release formulations such as LCPT (LCP-tacrolimus), which use a melt-dose technology to increase bioavailability and flatten peak–trough fluctuations, allowing similar exposure at approximately 20–30% lower total daily doses and lower peak concentrations (C_max) (9). In clinical practice, these pharmacokinetic advantages are frequently cited as a reason to switch patients from IR-tacrolimus to LCPT when neurotoxicity (particularly tremor or neurocognitive symptoms) is suspected to be driven by high peak concentrations. Nevertheless, the supporting evidence is limited and mixed; a Delphi consensus survey of expert clinicians still considered conversion from IR-tacrolimus to LCPT a potentially appropriate option for patients with troublesome neurocognitive adverse effects, while acknowledging the modest and somewhat controversial nature of the data underlying this recommendation (10).

Spontaneous reporting systems such as the FDA Adverse Event Reporting System (FAERS) provide large, real-world datasets to examine drug safety. By aggregating reports from multiple sources, FAERS enables detection of disproportionate reporting signals for rare or serious adverse events. Although it cannot estimate incidence or establish causality, FAERS is well suited to describing the spectrum of reported neuropsychiatric events and comparing relative reporting patterns between drugs. Importantly, serious outcomes reported in FAERS, including death, must be interpreted within the broader clinical context of transplant recipients and should not be assumed to reflect mortality directly attributable to neuropsychiatric toxicity alone.

Despite the widespread use of CNIs and recognition of their neurologic toxicity, pharmacovigilance studies specifically focusing on neuropsychiatric adverse events (AEs) with tacrolimus—including newer once-daily formulations such as LCPT—and cyclosporine remain limited. We therefore conducted a ten-year FAERS analysis to characterize CNI-associated neuropsychiatric events, quantify disproportionality signals for overall and specific neuropsychiatric phenotypes, and compare tacrolimus (across available oral formulations, including LCPT where identifiable) and cyclosporine with respect to the relative reporting of neuropsychiatric toxicity, including serious outcomes.

## Materials and methods

This retrospective pharmacovigilance study assessed reports of neuropsychiatric AEs related to CNI among transplant recipients, utilizing data from the FAERS. FAERS functions as a spontaneous reporting system designed for post-marketing safety surveillance and comprises reports submitted by healthcare professionals, consumers, and manufacturers. Given that FAERS is a passive reporting database, it is not suitable for estimating incidence rates or establishing causality; instead, it offers insights into reporting trends and potential safety signals. The FAERS Public Dashboard was examined for the period from January 1, 2015, to December 1, 2025, representing the complete timeframe captured within the downloaded datasets. Three drug-specific datasets were extracted based on the suspect medication: tacrolimus immediate-release (IR), LCPT, and cyclosporine. Each dataset contains all cases in which the drug was listed as a suspect agent. All downloaded files contained the core variables from FAERS, including Case ID, the latest FDA received date, event date, reporter information, patient demographics, outcomes, and MedDRA-coded reaction terms. De-duplication was performed independently for each drug-specific dataset in accordance with FDA-recommended FAERS data handling procedures. When multiple records shared the same Case ID, only the version with the most recent Latest FDA Received Date was retained, thereby preserving the most current case information and minimizing duplicate reporting. After de-duplication, a new variable (Drug_group) was created to classify each record as tacrolimus IR, LCPT (Envarsus), or cyclosporine. The three cleaned datasets were then merged into a consolidated master dataset with harmonized variable structures. Only reports from 2015 through 2025 were included in the final analytic dataset.

Because CNIs are used in multiple clinical settings, an additional restriction was applied to include only transplant-related AEs. The FAERS variable “Reason for Use” was utilized to identify cases explicitly associated with transplantation. Reports were included if the indication text contained terms referring to organ transplantation, including but not limited to adult and pediatric kidney/renal transplant, liver transplant, lung transplant, heart transplant, pancreas transplant, multiorgan transplant, prevention of rejection, or treatment of rejection. String-matching filters that are case-insensitive were applied. Reports lacking any transplant-related indication were excluded from subsequent analysis. The resulting dataset was defined as the transplant analytic cohort and served as the denominator population for both descriptive analyses and disproportionality testing. Additionally, reports were included if they met all of the following criteria: (1) one of the study drugs was listed as a suspect agent, (2) the report date fell within the prespecified study period, and (3) the indication text supported a transplant-related use. For the neuropsychiatric-specific analyses, cases were further required to contain at least one neurological or psychiatric Preferred Term as defined by the predefined MedDRA classification framework.

All reported reactions in FAERS are systematically coded using the Medical Dictionary for Regulatory Activities (MedDRA) Preferred Terms (PTs). To ensure consistent classification of neuropsychiatric AEs, a comprehensive dictionary was developed based on definitions from the Common Terminology Criteria for Adverse Events (CTCAE) version 5.0. Two PT lists were compiled, a neurological AE list and a psychiatric AE list, each mapped to CTCAE v5 definitions and derived from the Nervous System Disorders and Psychiatric Disorders sections. The neurological AE list included events such as tremor, headache, seizure, encephalopathy, stroke/transient ischemic attack (TIA), peripheral neuropathy, dizziness, ataxia, paresthesia, syncope, and cognitive disturbance. The psychiatric AE list contains depression, anxiety, insomnia, agitation, irritability, hallucinations, delirium, psychosis, and suicidal ideation. Any report containing at least one PT from these lists was flagged as either Neuro_AE, Psych_AE, or Both. A mutually exclusive variable (AE_Category) was established to classify each report into one of the following categories: (1) neurological only, (2) psychiatric only, (3) both neurological and psychiatric, or (4) none. Only categories 1 through 3 were included in the neuropsychiatric analyses and summary tables. For consistency, the terms neurological, psychiatric, and combined neuropsychiatric refer to the predefined analytic categories used in this study, whereas individual terms such as delirium, encephalopathy, psychosis, anxiety, and insomnia refer to specific MedDRA Preferred Terms or clinically distinct symptom constructs. The complete list of neurological and psychiatric MedDRA Preferred Terms used for classification is provided in **Supplementary Table 1**.

Several FAERS variables were harmonized before analysis to ensure consistency across the three datasets. The Latest FDA Received Date was converted into both calendar year and calendar quarter (Q1–Q4) to enable evaluation of temporal reporting patterns across the study period (2015–2025). Age data were cleaned and grouped into predefined categories (<18, 18–34, 35–49, 50–64, 65–74, ≥75, and Unknown), while retaining continuous age for summary purposes. Sex was standardized into Female, Male, and Unknown. Reporter type was consolidated into Healthcare Professional, Consumer, and Unknown, based on occupation fields. The country of event occurrence was harmonized into the United States, Non-US, and Unknown. Seriousness was assessed against FDA criteria (death, life-threatening condition, hospitalization, disability, congenital anomaly, required intervention, or other medically significant condition), with individual components and a binary Serious/Non-Serious indicator created. All these standardized variables were integrated into a unified dataset and used consistently across descriptive, neuropsychiatric AE classification, temporal trend analysis, and inference procedures.

Descriptive statistics were employed to characterize neuropsychiatric adverse event reporting across the three drug groups. Categorical variables were summarized as frequencies and percentages, while continuous variables were described using medians and interquartile ranges. The standardized dataset facilitated the comparison of demographic characteristics, adverse event categories, Preferred Term frequencies, and seriousness outcomes across tacrolimus IR, LCPT, and cyclosporine. Temporal patterns in neuropsychiatric reporting from 2015 to 2025 were evaluated using the derived calendar-year variable, and additional visualizations were generated to illustrate overall and drug-specific reporting trends. To identify disproportional reporting, disproportionality analyses were performed using the Reporting Odds Ratio (ROR) and Proportional Reporting Ratio (PRR). The primary criterion for elevated disproportionality was a lower bound of the 95% confidence interval for the ROR greater than 1, with at least 3 reports for the drug–event pair. PRR values and corresponding 95% confidence intervals were calculated as supportive secondary measures. Because several highlighted associations did not meet the conventional PRR threshold of ≥2, PRR was not used as the sole determinant of signal detection in the present analysis. For each drug–event pair, 2×2 contingency tables were constructed within the transplant-restricted cohort, comparing the drug of interest with the other study drugs combined rather than with the full FAERS database. In this framework, a represents reports with the event for the drug of interest, b reports without the event for the drug of interest, c reports with the event for the comparator drugs, and d reports without the event for the comparator drugs. ROR was calculated as (a/c)/(b/d), equivalent to ad/bc, and PRR was calculated as [a/(a+b)]/[c/(c+d)]. These analyses were conducted in accordance with established FAERS signal detection conventions. All analyses were performed according to the predefined analysis plan using SAS software (version 9.4; SAS Institute Inc., Cary, NC, USA).

## Results

Between 2015 and 2025, a total of 5,437 neuropsychiatric adverse event reports involving transplant recipients treated with CNIs were identified in FAERS. Most reports involved tacrolimus immediate-release (tacrolimus IR; n = 3,909; 71.9%), followed by cyclosporine (n = 1,286; 23.7%) and LCPT (once-daily extended-release tacrolimus; n = 242; 4.5%). Neuropsychiatric AE categories are summarized in **Table 1**. Neurological-only events constituted 74.1% of reports, psychiatric-only 17.1%, and combined neuropsychiatric events 8.8%. Sex distribution was similar across drugs (48.3% male, 43.3% female, 8.5% unknown). Tacrolimus IR reports predominantly involved adults aged 50–64 years (31.0%), whereas cyclosporine had a higher proportion of pediatric cases (<18 years: 17.8%). The overall median age was 52 years (IQR 37–62). Most reports originated from consumers (83.7%) rather than healthcare professionals (16.3%) and from non-US regions (68.1%). Serious outcomes were common: 92.5% of reports were classified as serious, with hospitalization reported in 52.1% and death in 16.2% (**Table 1**).

**Table 1:** Baseline characteristics of transplant patients with neuropsychiatric adverse event reports in FAERS.

Characteristic	Tacrolimus immediate release	LCP-tacrolimus	Cyclosporine	Total
Number of AE reports, N	3909	242	1286	5437
AE Category				
Neurological-only	2958 (75.7%)	182 (75.2%)	888 (69.1%)	4028 (74.1%)
Psychiatric-only	622 (15.9%)	39 (16.1%)	268 (20.8%)	929 (17.1%)
Combined neuropsychiatric	329 (8.4%)	21 (8.7%)	130 (10.1%)	480 (8.8%)
Sex				
Female	1647 (42.1%)	111 (45.9%)	594 (46.2%)	2352 (43.3%)
Male	1922 (49.2%)	123 (50.8%)	580 (45.1%)	2625 (48.3%)
Unknown Sex	340 (8.7%)	8 (3.3%)	112 (8.7%)	460 (8.5%)
Age group				
<18	315 (8.1%)	4 (1.7%)	229 (17.8%)	548 (10.1%)
18–34	360 (9.2%)	15 (6.2%)	129 (10.0%)	504 (9.3%)
35–49	673 (17.2%)	22 (9.1%)	248 (19.3%)	943 (17.3%)
50–64	1211 (31.0%)	92 (38.0%)	339 (26.4%)	1642 (30.2%)
65–74	559 (14.3%)	45 (18.6%)	97 (7.5%)	701 (12.9%)
≥75	116 (3.0%)	17 (7.0%)	16 (1.2%)	149 (2.7%)
Unknown Age	675 (17.3%)	47 (19.4%)	228 (17.7%)	950 (17.5%)
Reporter Type				
Healthcare professional	748 (19.1%)	30 (12.4%)	106 (8.2%)	884 (16.3%)
Consumer	3161 (80.9%)	212 (87.6%)	1180 (91.8%)	4553 (83.7%)
Unknown	0 (0.0%)	0 (0.0%)	0 (0.0%)	0 (0.0%)
Country				
US	1243 (31.8%)	86 (35.5%)	152 (11.8%)	1481 (27.2%)
Non-US	2480 (63.4%)	137 (56.6%)	1086 (84.4%)	3703 (68.1%)
Unknown Country	186 (4.8%)	19 (7.9%)	48 (3.7%)	253 (4.7%)
Seriousness				
Serious	3563 (91.1%)	209 (86.4%)	1256 (97.7%)	5028 (92.5%)
Non-serious	346 (8.9%)	33 (13.6%)	30 (2.3%)	409 (7.5%)
Outcomes				
Death	591 (15.1%)	26 (10.7%)	266 (20.7%)	883 (16.2%)
Life-threatening	513 (13.1%)	22 (9.1%)	206 (16.0%)	741 (13.6%)
Hospitalization	2127 (54.4%)	129 (53.3%)	577 (44.9%)	2833 (52.1%)
Disability	69 (1.8%)	6 (2.5%)	20 (1.6%)	95 (1.7%)
Other serious	2629 (67.3%)	149 (61.6%)	940 (73.1%)	3718 (68.4%)
Median Age (IQR)	53.0 (39.0-63.0)	61.0 (53.0-67.0)	45.0 (21.0-59.0)	52.0 (37.0-62.0)

Neurological manifestations are summarized in **Table 2**. Across all three medications, encephalopathy was the most frequently reported neurological AE, present in 34.8% of neurological AE reports overall (31.2% for tacrolimus IR, 27.6% for LCPT, and 47.7% for cyclosporine). Tremor and headache were also common, each accounting for approximately 20.3% of neurological AEs in the overall cohort. Seizures were reported with all three agents, comprising 15.0% of neurological AEs overall (13.7% for tacrolimus IR, 9.9% for LCPT, and 20.1% for cyclosporine). Neuropathy was less frequent but observed with each drug (1.5–6.6%). Overall, the most frequently reported neurological AEs across the three study drugs were encephalopathy, tremor, headache, and seizures (**Table 2**). Psychiatric AEs are summarized in **Table 3**. Insomnia was the most frequently reported psychiatric event, accounting for 31.6% of psychiatric AEs overall (28.7% for tacrolimus IR, 25.0% for LCPT, and 39.4% for cyclosporine). Mood-related symptoms were also prominent: depression occurred in 12.3% and anxiety in 9.0% of psychiatric AEs overall, and were reported with all three medications. Hallucinations (7.7% overall) and psychosis (1.3%) were less common but were observed across all drugs except psychosis with LCPT, for which no cases were reported. Thus, psychiatric AEs under CNI therapy were dominated by insomnia, depression, and anxiety, with additional contributions from hallucinations and psychosis (**Table 3**).

**Table 2:** Most frequently reported neurological adverse events in transplant patients for Neuro=1.

Neurological AE (PT)	Tacrolimus immediate release	LCP-tacrolimus	Cyclosporine	Total
AE_Headache	703 (21.4%)	35 (17.2%)	179 (17.6%)	917 (20.3%)
AE_Tremor	752 (22.9%)	77 (37.9%)	88 (8.6%)	917 (20.3%)
AE_Seizure	449 (13.7%)	20 (9.9%)	205 (20.1%)	674 (15.0%)
AE_Encephalopathy	1025 (31.2%)	56 (27.6%)	486 (47.7%)	1567 (34.8%)
AE_Neuropathy	51 (1.6%)	3 (1.5%)	67 (6.6%)	121 (2.7%)

**Table 3:** Most frequently reported psychiatric adverse events in transplant patients for Psych AE = 1.

Psych AE (PT)	Tacrolimus immediate release	LCP-tacrolimus	Cyclosporine	Total
AE_Anxiety	79 (8.3%)	8 (13.3%)	40 (10.1%)	127 (9.0%)
AE_Depression	116 (12.2%)	9 (15.0%)	48 (12.1%)	173 (12.3%)
AE_Insomnia	273 (28.7%)	15 (25.0%)	157 (39.4%)	445 (31.6%)
AE_Hallucination	73 (7.7%)	6 (10.0%)	29 (7.3%)	108 (7.7%)
AE_Psychosis	11 (1.2%)	0 (0.0%)	8 (2.0%)	19 (1.3%)

Clinical outcomes associated with neuropsychiatric AEs are presented in **Table 4**. Among neurological-only events, hospitalization was the most frequent serious outcome, reported in 54.7% of tacrolimus IR, 51.6% of LCPT, and 44.3% of cyclosporine cases (overall 52.3%). “Other serious” outcomes, as defined by FAERS (medically important conditions), were also common, affecting 68.8% of neurological-only reports overall. For psychiatric-only AEs, hospitalization occurred in 46.3% and other serious outcomes in 65.0%. Life-threatening outcomes were proportionally more frequent among psychiatric-only AEs (29.2%) than neurological-only events (10.6%). In reports with combined neuropsychiatric events, hospitalization was highest among all categories (62.1%) and other serious outcomes were reported in 71.7%. Death was documented across all categories, with the highest proportions among neurological-only (16.8%) and combined neuropsychiatric (16.5%) events. Collectively, neuropsychiatric AE reports involving tacrolimus IR, LCPT, and cyclosporine frequently included serious outcomes (**Table 4**). Yearly reporting trends are shown in **Table 5**. Total neuropsychiatric AE reports increased from 322 in 2015 to a peak of 727 in 2019, and then remained elevated through 2020–2025 (range 344–710 reports per year). Tacrolimus IR consistently accounted for the largest share, increasing from 233 reports in 2015 to 533 in 2019, and remaining between 250 and 506 reports annually thereafter. LCPT-related reports were fewer in absolute number but gradually increased over time, reaching 36 in 2025. Cyclosporine-related reports rose from 89 in 2015 to 173 in 2025, with an earlier peak in 2019 and renewed increases in 2024–2025. Overall, neuropsychiatric AE reporting for CNIs remained sustained over the latter half of the study period. These descriptive temporal patterns may reflect changes in reporting behavior, clinical awareness, and prescribing patterns over time, and should not be interpreted as direct evidence of changing incidence.

**Table 4:** Clinical outcomes associated with neuropsychiatric adverse events by drug and AE category.

Outcome	AE Category	Tacrolimus immediate release n (%)	LCP-tacrolimus n (%)	Cyclosporine n (%)	Total n (%)
Death	Neurological-only	470 (15.9%)	16 (8.8%)	191 (21.5%)	677 (16.8%)
	Psychiatric-only	83 (13.3%)	9 (23.1%)	35 (13.1%)	127 (13.7%)
	Combined neuropsychiatric	38 (11.6%)	1 (4.8%)	40 (30.8%)	79 (16.5%)
Life-threatening	Neurological-only	336 (11.4%)	17 (9.3%)	75 (8.4%)	428 (10.6%)
	Psychiatric-only	149 (24.0%)	3 (7.7%)	119 (44.4%)	271 (29.2%)
	Combined neuropsychiatric	28 (8.5%)	2 (9.5%)	12 (9.2%)	42 (8.8%)
Hospitalization	Neurological-onlyNeurological-only	1618 (54.7%)	94 (51.6%)	393 (44.3%)	2105 (52.3%)
	Psychiatric-only	302 (48.6%)	24 (61.5%)	104 (38.8%)	430 (46.3%)
	Combined neuropsychiatric	207 (62.9%)	11 (52.4%)	80 (61.5%)	298 (62.1%)
Disability	Neurological-onlyNeurological-only	60 (2.0%)	6 (3.3%)	16 (1.8%)	82 (2.0%)
	Psychiatric-only	5 (0.8%)	0 (0.0%)	1 (0.4%)	6 (0.6%)
	Combined neuropsychiatric	4 (1.2%)	0 (0.0%)	3 (2.3%)	7 (1.5%)
Other Serious	Neurological-onlyNeurological-only	1997 (67.5%)	115 (63.2%)	658 (74.1%)	2770 (68.8%)
	Psychiatric-only	402 (64.6%)	24 (61.5%)	178 (66.4%)	604 (65.0%)
	Combined neuropsychiatric	230 (69.9%)	10 (47.6%)	104 (80.0%)	344 (71.7%)

**Table 5:** Yearly trends in neuropsychiatric adverse event reporting among transplant patients.

Year	Tacrolimus immediate release	LCP-tacrolimus	Cyclosporine	Total
2015	233	0	89	322
2016	263	6	70	339
2017	367	11	138	516
2018	494	28	130	652
2019	533	47	147	727
2020	506	39	165	710
2021	324	29	84	437
2022	314	15	73	402
2023	250	10	84	344
2024	315	21	133	469
2025	310	36	173	519

**Figure 1:**
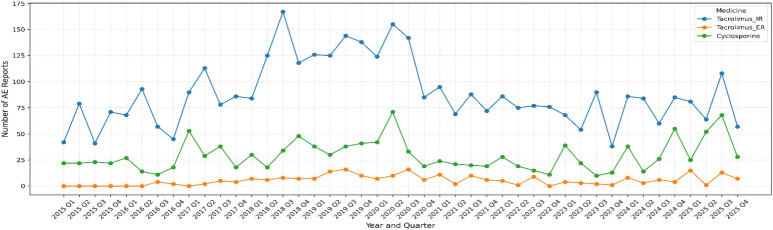
Quarterly reporting trends of neuropsychiatric adverse events associated with tacrolimus immediate-release (tacrolimus_IR), LCP tacrolimus (LCPT), and cyclosporine from 2015 through 2025 in the FAERS database. The figure illustrates the number of neuropsychiatric AE reports submitted each calendar quarter, demonstrating sustained reporting activity across all three agents, with tacrolimus_IR consistently accounting for the largest proportion of quarterly submissions.

Quarterly patterns mirrored the annual trends (**Figure 1**). Tacrolimus IR generated the highest number of neuropsychiatric AE reports in nearly every quarter, with a marked rise through 2018–2019 and persistent, though variable, reporting from 2020–2025. Cyclosporine showed a moderate but stable quarterly reporting pattern with intermittent peaks, whereas LCPT had the lowest absolute quarterly volume but a gradual upward trajectory. Together, these findings provide temporal context for interpreting the observed disproportionality patterns across the three CNI groups.

**Figure 2:**
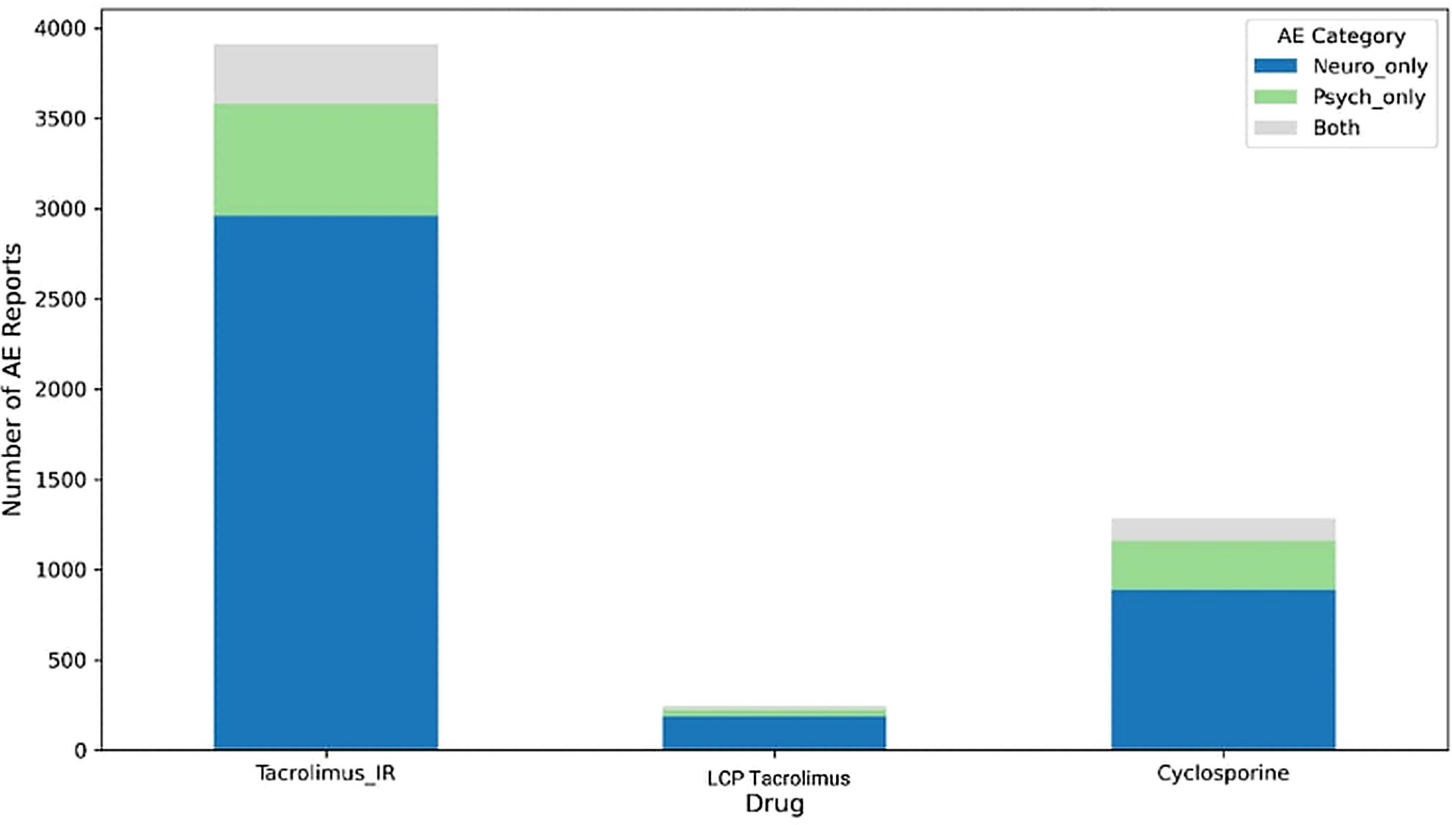
Stacked bar chart showing the distribution of neuropsychiatric adverse event (AE) categories— neuro only, psych-only, and combined neuropsychiatric events (“both”)—for tacrolimus immediate-release (tacrolimus_IR), LCP tacrolimus (LCPT), and cyclosporine among transplant recipients in the FAERS database. Neuro only AEs accounted for the majority of reports across all three medications, with smaller contributions from psych-only and combined AE categories.

The distribution of neuropsychiatric AE categories by drug is shown in **Figure 2**. For tacrolimus IR, LCPT, and cyclosporine, neurological-onlyevents consistently comprised the majority of reports, with smaller proportions of psychiatric-only and combined neuropsychiatric events. Tacrolimus IR had the highest absolute number of neurological-onlyAEs, whereas cyclosporine displayed a similar relative distribution despite lower total counts. LCPT followed the same pattern, with neurological-onlyAEs predominating and psychiatric-only and combined events forming smaller fractions. Thus, despite differences in total reporting volume, the relative distribution of AE categories was broadly similar across the three CNIs (**Figure 2**).

**Figure 3:**
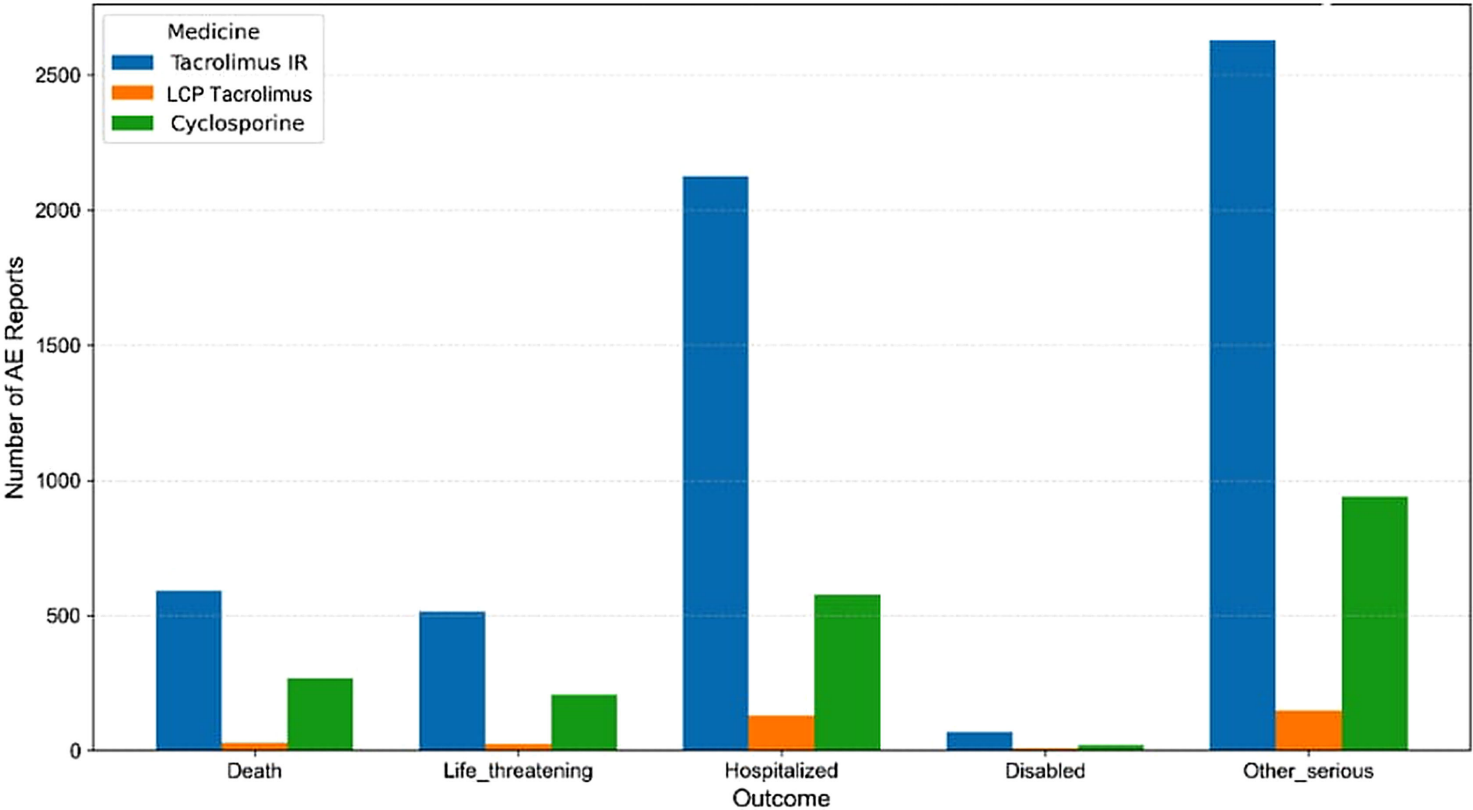
Clinical outcomes of neuropsychiatric AE reports. Among all three calcineurin inhibitors, hospitalization and “other serious” outcomes made up the largest share of reported neuropsychiatric adverse events, particularly with tacrolimus IR, where more than half of reports involved hospitalization (n = 2,127) and about two-thirds were classified as other serious outcomes (n = 2,629). Death and life-threatening events were less common but still notable, with 591 deaths and 513 life-threatening events reported for tacrolimus IR, 26 and 22 respectively for LCP tacrolimus, and 266 and 206 for cyclosporine. Cyclosporine showed a relatively higher proportion of death and life-threatening outcomes compared with the tacrolimus formulations, while disability remained rare for all three drugs (≤2–3% of reports).

Aggregated serious outcomes by drug are depicted in **Figure 3**. Across all three CNIs, hospitalization and “other serious” outcomes comprised the largest share of reported neuropsychiatric AEs. For tacrolimus IR, more than half of reports involved hospitalization (n = 2,127) and approximately two-thirds were classified as other serious outcomes (n = 2,629). Death and life-threatening events were less frequent but clinically significant, with 591 deaths and 513 life-threatening events reported for tacrolimus IR, 26 and 22 for LCPT, and 266 and 206 for cyclosporine, respectively. Relative to tacrolimus formulations, cyclosporine showed a higher proportion of death and life-threatening outcomes, whereas disability was uncommon for all three agents (≤2–3% of reports).

Disproportionality results for selected neurological and psychiatric PTs are summarized in **Table 6**. For tacrolimus IR, elevated reporting disproportionality was observed for tremor (ROR 1.968, 95% CI 1.643–2.356; PRR 1.782, 95% CI 1.521–2.086) and, to a lesser extent, headache (ROR 1.346, 95% CI 1.141–1.589; PRR 1.284, 95% CI 1.115–1.479). No meaningful ROR-based disproportionality was observed for encephalopathy, insomnia, anxiety, or depression. For LCPT, tremor showed the highest ROR-based disproportionality (ROR 2.419, 95% CI 1.828–3.203; PRR 1.968, 95% CI 1.620–2.390), whereas no other selected PT showed elevated disproportionality. In contrast, cyclosporine showed elevated reporting disproportionality for encephalopathy (ROR 1.725, 95% CI 1.511–1.969; PRR 1.451, 95% CI 1.330–1.583), insomnia (ROR 1.865, 95% CI 1.519–2.291; PRR 1.760, 95% CI 1.464–2.115), and anxiety (ROR 1.500, 95% CI 1.026–2.192; PRR 1.484, 95% CI 1.026–2.147), whereas tremor and headache did not show elevated disproportionality. Taken together, these findings suggest distinct drug-specific neuropsychiatric reporting patterns, with both tacrolimus formulations showing greater disproportionality for tremor reporting, whereas cyclosporine showed greater disproportionality for encephalopathy, insomnia, and anxiety.

**Table 6:** Disproportionality analysis (ROR and PRR) for selected neurological and psychiatric Preferred Terms associated with tacrolimus IR, LCPT, and cyclosporine.

Tacrolimus immediate release		
AE	ROR (95% CI)	PRR (95% CI)
Neurological Preferred Terms		
AE_Encephalopathy	0.647 (0.57–0.734)	0.739 (0.679–0.805)
AE_Tremor	1.968 (1.643–2.356)	1.782 (1.521–2.086)
AE_Headache	1.346 (1.141–1.589)	1.284 (1.115–1.479)
Psychiatric Preferred Terms		
AE_Insomnia	0.592 (0.484–0.724)	0.62 (0.517–0.744)
AE_Depression	0.789 (0.572–1.09)	0.796 (0.583–1.086)
AE_Anxiety	0.636 (0.442–0.915)	0.643 (0.452–0.916)
LCP-tacrolimus		
AE	ROR (95% CI)	PRR (95% CI)
Neurological Preferred Terms		
AE_Encephalopathy	0.734 (0.541–0.996)	0.796 (0.63–1.005)
AE_Tremor	2.419 (1.828–3.203)	1.968 (1.62–2.39)
AE_Headache	0.827 (0.574–1.192)	0.852 (0.623–1.164)
Psychiatric Preferred Terms		
AE_Insomnia	0.732 (0.43–1.246)	0.749 (0.455–1.233)
AE_Depression	1.185 (0.598–2.348)	1.178 (0.61–2.276)
AE_Anxiety	1.458 (0.704–3.019)	1.443 (0.714–2.918)
Cyclosporine		
AE	ROR (95% CI)	PRR (95% CI)
Neurological Preferred Terms		
AE_Encephalopathy	1.725 (1.511–1.969)	1.451 (1.33–1.583)
AE_Tremor	0.294 (0.234–0.37)	0.343 (0.278–0.423)
AE_Headache	0.748 (0.627–0.892)	0.783 (0.673–0.91)
Psychiatric Preferred Terms		
AE_Insomnia	1.865 (1.519–2.291)	1.76 (1.464–2.115)
AE_Depression	1.249 (0.89–1.752)	1.239 (0.894–1.719)
AE_Anxiety	1.5 (1.026–2.192)	1.484 (1.026–2.147)

## Discussion

In this large pharmacovigilance analysis of FAERS reports from 2015–2025, neuropsychiatric AEs associated with CNIs in transplant recipients were commonly reported and predominantly serious. Among 5,437 transplant-related neuropsychiatric reports, tacrolimus IR accounted for 71.9%, cyclosporine for 23.7%, and LCPT for 4.5%. Overall, 92.5% of reports were classified as serious, more than half involved hospitalization (52.1%), and death was reported in 16.2% of cases. These seriousness outcomes warrant cautious interpretation, as transplant recipients represent a clinically complex population in whom hospitalization and death are often driven by multifactorial illness rather than by CNI-related neuropsychiatric toxicity alone. For example, in a kidney transplant cohort, infection has accounted for approximately 69.6% of deaths and cardiovascular causes for about 12.7%, supporting the view that mortality in this setting is usually driven by broader clinical illness rather than isolated CNI-related neuropsychiatric toxicity (11). In parallel, CNI-related neurotoxicity is generally described as dose-dependent and often reversible with dose reduction, discontinuation, or conversion of therapy (12). Disproportionality analyses identified drug-specific reporting patterns: tremor showed elevated disproportionality for both tacrolimus formulations (tacrolimus IR ROR 1.97, 95% CI 1.64–2.36; LCPT ROR 2.42, 95% CI 1.83–3.20), whereas encephalopathy, insomnia, and anxiety showed elevated disproportionality for cyclosporine (RORs ~1.5–1.9); other neuropsychiatric preferred terms did not consistently show elevated ROR-based disproportionality. These findings extend prior clinical data on CNI-associated neurotoxicity and provide a large real-world comparison that, importantly, includes once-daily LCPT. However, they should be interpreted as signals of disproportionate reporting within a spontaneous pharmacovigilance database rather than as evidence of causation, comparative risk, or true incidence. This distinction is especially relevant given that tacrolimus is generally regarded as the more effective CNI for prevention of acute rejection and is therefore more commonly used than cyclosporine in many solid organ transplant settings (13, 14). This greater use may partly contribute to the higher absolute number of tacrolimus-related reports in our dataset. Because FAERS lacks treatment denominators, a larger number of tacrolimus reports should not be interpreted as evidence of a higher incidence of neuropsychiatric toxicity, but may instead reflect its broader use in contemporary transplant practice.

Neurotoxicity of CNIs has been recognized for decades, with reported prevalence in solid organ transplant cohorts commonly ranging from 10% to 30%, and in some series up to 40% for tacrolimus-treated recipients (12, 15–17). The clinical spectrum includes minor manifestations such as tremor, headache, and insomnia, as well as major events such as seizures, toxic encephalopathy, and PRES (18, 19). Additionally, attribution of CNI neurotoxicity is typically supported by the broader clinical context, including dose or exposure relationships, serum drug concentrations, temporal association with treatment initiation or dose escalation, and reversibility after dose reduction, discontinuation, or conversion to an alternative regimen (12). In comparative studies, tacrolimus has been associated with higher rates of neurologic complications than cyclosporine, particularly tremor, paresthesia, and insomnia (18, 20, 21). A recent systematic review of tacrolimus-induced neurotoxicity estimated overall neurotoxicity (including tremor, headache, and seizures) in approximately 30% of tacrolimus-treated patients (14). These data are consistent with our pharmacovigilance findings: tremor showed robust disproportionality for tacrolimus IR and LCPT, whereas cyclosporine did not demonstrate a tremor signal and instead was disproportionately associated with encephalopathy and anxiety/insomnia. The convergence between clinical and pharmacovigilance evidence is consistent with tremor is a characteristic, drug-specific manifestation of tacrolimus neurotoxicity, while cyclosporine more often presents with encephalopathic and affective/sleep phenotypes.

The LCPT-specific observations are particularly relevant to contemporary practice. Formulation-specific pharmacokinetics may provide a biologically plausible context for differences in neuropsychiatric reporting patterns, but these data should be interpreted cautiously. Tacrolimus IR and LCPT differ in absorption characteristics, bioavailability, and peak-to-trough fluctuation, which may influence tolerability in some clinical settings. LCPT, through MeltDose^®^ technology, is designed to increase tacrolimus bioavailability and produce a flatter concentration–time profile, allowing similar or greater exposure at approximately 20–30% lower total daily doses with reduced peak concentrations and less peak-to-trough fluctuation (17). However, the formulation-specific reporting differences observed in FAERS should not be interpreted as evidence of causally distinct neuropsychiatric risk profiles. Rather, they should be viewed as hypothesis-generating observations that may be compatible with known pharmacokinetic differences but remain confounded by indication, channeling, clinical severity, and other unmeasured factors. Consistent with the pharmacologic rationale for smoother exposure, the STRATO phase 3b trial in 44 kidney transplant recipients with clinically significant tacrolimus-related tremor found that switching from twice-daily tacrolimus to once-daily LCPT led to statistically and clinically meaningful reductions in tremor severity within 7 days, with parallel improvements in tremor-related quality of life (16). Subsequent observational cohorts, including the ELIT study, similarly reported sustained reductions in tremor scores, improved quality of life, stable graft function, and favorable C_0_/D ratios after conversion to LCPT (17). More recent data in both kidney and liver transplantation have likewise described reduced tremor burden and favorable pharmacokinetic profiles after early or late conversion to LCPT (17, 22). Against this background, the tremor signal we observed for LCPT (ROR 2.42; proportional reporting ratio 1.97) should not be interpreted as evidence that LCPT inherently confers higher tremor reporting disproportionality than IR tacrolimus. FAERS does not capture symptom trajectories or changes after a switch; it only records that tremor occurred at some point while LCPT was a suspect drug. Given that LCPT is frequently prescribed specifically to patients with problematic tacrolimus-related tremor, confounding by indication is highly likely (16, 17, 22). Statistically, the elevated ROR for LCPT therefore more plausibly reflects selective channeling of high-risk patients to this formulation rather than a formulation-driven increase in tremor incidence.

The cyclosporine profile in our analysis was dominated by encephalopathy (ROR 1.73, 95% CI 1.51–1.97), insomnia (ROR 1.87, 95% CI 1.52–2.29), and anxiety (ROR 1.50, 95% CI 1.03–2.19), with no tremor or headache signal. CNI-associated encephalopathy and PRES have been well documented in both solid organ and hematopoietic stem cell transplantation, with cyclosporine historically featuring prominently in early descriptions (23, 24). Proposed mechanisms include endothelial injury, disruption of the blood–brain barrier, and impaired cerebral autoregulation, often in the setting of hypertension, renal dysfunction, and high CNI exposure (12). Reviews of CNI neurotoxicity emphasize that psychiatric manifestations—insomnia, anxiety, mood changes, hallucinations, and psychosis—are increasingly recognized but likely under-reported in routine care (21). The pattern observed in our dataset, in which cyclosporine is disproportionately more frequently reported with encephalopathy and insomnia/anxiety whereas tacrolimus formulations are dominated by tremor, suggests that differences in pharmacokinetic characteristics, CNS penetration, and vascular effects may contribute to differing neuropsychiatric reporting patterns at the population level.

The gradients in seriousness across AE categories are also noteworthy. Psychiatric-only reports in our cohort had a higher proportion of life-threatening outcomes than neurological-onlyevents (29.2% vs 10.6%), despite representing a smaller fraction of total reports, and combined neuropsychiatric events had the highest hospitalization rate (62.1%). While reporting bias almost certainly contributes—severe and dramatic presentations are more likely to be reported—these patterns suggest that psychiatric manifestations of CNI toxicity, particularly when accompanied by neurologic signs, may mark a subgroup of patients with greater overall medical instability or multisystem involvement. This is consistent with case series where CNI-related psychosis or delirium often co-occurs with metabolic derangements, infections, or cerebrovascular complications (12, 15).

From a clinical standpoint, these findings have several implications. First, the very high proportion of serious outcomes and the observed mortality underscore the need for a low threshold to investigate new or worsening neurologic and psychiatric symptoms in CNI-treated transplant recipients. Structured screening for tremor, cognitive changes, visual disturbances, seizures, insomnia, and mood or anxiety symptoms should be incorporated into routine post-transplant follow-up. At the same time, the reported FAERS outcome of death should not be interpreted as evidence that the CNI caused death. Rather, it indicates that death was reported in a case in which the drug was listed as a suspect product. In transplant recipients, death may reflect underlying illness severity, graft-related complications, infection, malignancy, cardiovascular events, concomitant medications, or other clinical factors independent of the reported neuropsychiatric AE or the CNI itself. Accordingly, deaths in this dataset should be interpreted as occurring during treatment exposure or case reporting context, not as CNI-attributable mortality. Second, our quantitative results support current practice patterns that use formulation conversion as a targeted strategy for tremor-predominant tacrolimus toxicity. Randomized and observational studies consistently show significant, clinically meaningful reductions in tremor and improved quality of life after switching from IR tacrolimus to LCPT, while maintaining immunosuppressive efficacy and stable graft outcomes (16, 17, 19). Third, the cyclosporine signals for encephalopathy and insomnia/anxiety argue for heightened vigilance when this agent is used in patients with pre-existing cerebrovascular risk, uncontrolled hypertension, or psychiatric vulnerability, and they provide quantitative support for considering conversion to alternative CNIs or non-CNI regimens (such as belatacept) in patients with recurrent or severe encephalopathic events, as suggested by case reports and small series (23, 24). Notably, in clinical practice, tacrolimus-associated PRES frequently leads to conversion from tacrolimus to cyclosporine as an acute management strategy, reflecting historical experience that cyclosporine may, in selected cases, be better tolerated from a neurovascular standpoint during recovery from PRES.

This study has the inherent limitations of spontaneous reporting data. FAERS lacks exposure denominators; therefore, ROR and PRR reflect relative reporting rather than incidence among treated patients, and these analyses cannot estimate absolute event frequency or comparative risk across therapies. Under-reporting is substantial and non-random, and reporting intensity may vary over time and between drugs. Accordingly, the observed temporal patterns may reflect changes in reporting behavior, clinical awareness, formulation uptake, or prescribing practices rather than true changes in underlying event incidence. Interpretation is further limited by the clinical complexity of transplant recipients, in whom neuropsychiatric syndromes are often multifactorial and may reflect infection, metabolic derangements, corticosteroid exposure, renal dysfunction, graft dysfunction, critical illness, or baseline psychiatric comorbidity rather than a direct medication effect alone. In this setting, FAERS cannot reliably distinguish medication-induced neuropsychiatric syndromes from events driven by concurrent medical, neurologic, or psychological contributors. In addition, important clinical covariates—including CNI dose, trough concentrations, time since transplantation, graft type, and concomitant neurotoxic or psychotropic medications—are incompletely captured, limiting adjustment for confounding. FAERS also does not provide sufficiently granular and standardized information on organ-specific therapeutic targets, longitudinal drug levels, or timing after transplantation to support valid subgroup comparisons. Accordingly, our results should be interpreted as overall transplant-population pharmacovigilance signals rather than as evidence of uniform neuropsychiatric risk across individual organ types.Confounding by indication is particularly relevant for LCPT, which is often used in patients with pre-existing tacrolimus-related neurotoxicity. Misclassification of transplant status or neuropsychiatric phenotype is also possible despite standardized MedDRA coding. Finally, disproportionality findings cannot establish causality and should therefore be interpreted as hypothesis-generating signals requiring confirmation in randomized trials, prospective cohorts, and mechanistic studies.Notwithstanding these limitations, the analysis has several strengths: it encompasses an 10-year period; focuses specifically on transplant recipients; includes three clinically relevant CNI regimens, including LCPT; and systematically separates neurologic from psychiatric phenotypes while applying established pharmacovigilance methods. When integrated with the existing clinical and pharmacokinetic literature, our findings support a more individualized approach to CNI selection and modification in transplantation, in which neuropsychiatric tolerability is considered alongside immunologic efficacy, nephrotoxicity, and metabolic risk as a core dimension of long-term immunosuppressive management.

## Conclusions

In this 10-year FAERS analysis of transplant recipients treated with CNIs, neuropsychiatric adverse events were commonly reported and were predominantly classified as serious, with substantial proportions involving hospitalization and reported death. Tacrolimus IR accounted for most reports, although LCPT and cyclosporine also contributed. This predominance should be interpreted in the context of the broader use of tacrolimus in modern transplant practice.

Disproportionality analyses identified distinct drug-specific reporting patterns: both tacrolimus formulations showed elevated reporting disproportionality for tremor, whereas cyclosporine showed greater reporting disproportionality for encephalopathy, insomnia, and anxiety. These findings are broadly consistent with prior clinical literature and support the interpretation that both CNI type and formulation may contribute to differing neuropsychiatric reporting patterns. However, these patterns may also vary across organ types because therapeutic targets, time from transplantation, concomitant immunosuppression, and immunologic risk differ across transplant populations.

Given the inherent limitations of spontaneous reporting data, these findings should be interpreted as hypothesis-generating pharmacovigilance signals rather than evidence of causation, incidence, comparative risk or drug-attributable mortality. Nonetheless, they underscore the importance of considering neuropsychiatric tolerability when individualizing CNI selection and modification in clinical practice. Future work should integrate pharmacovigilance findings with transplant registries, therapeutic drug monitoring, and prospective clinical studies to better characterize formulation-specific neuropsychiatric safety patterns and improve individualized risk assessment.

## Data Availability

The raw data supporting the conclusions of this article will be made available by the authors, without undue reservation.

## Supplementary material

Supplementary Table 1 Predefined MedDRA Preferred Terms used for classification of neurological and psychiatric adverse events.
