# Supplementary material for: Comparative neuropsychiatric safety signals of tacrolimus versus cyclosporine in solid organ transplantation: ten-year FAERS pharmacovigilance study
Source: Front Immunol. 2026 Mar 27;17:1795626. doi: 10.3389/fimmu.2026.1795626 (PMC13065514; doi:10.3389/fimmu.2026.1795626)
Supplement: Supplementary Table 1 — Predefined MedDRA Preferred Terms used for classification of neurological and psychiatric adverse events. [file Table1.docx]

Supplementary Table S1. Predefined MedDRA Preferred Terms used for classification of neurological and psychiatric adverse events

| **Analytic category** | **MedDRA Preferred Term** |
| --- | --- |
| Neurological | Tremor |
| Neurological | Headache |
| Neurological | Seizure |
| Neurological | Encephalopathy |
| Neurological | Peripheral neuropathy |
| Neurological | Dizziness |
| Neurological | Ataxia |
| Neurological | Paresthesia |
| Neurological | Syncope |
| Neurological | Cognitive disturbance |
| Psychiatric | Insomnia |
| Psychiatric | Depression |
| Psychiatric | Anxiety |
| Psychiatric | Agitation |
| Psychiatric | Irritability |
| Psychiatric | Hallucination |
| Psychiatric | Delirium |
| Psychiatric | Psychosis |
| Psychiatric | Suicidal ideation |
